# Supplementary material for: Identification of photoperiod-induced specific miRNAs in the adrenal glands of Sunite sheep (Ovis aries)
Source: Front Vet Sci. 2022 Jul 22;9:888207. doi: 10.3389/fvets.2022.888207 (PMC9354845; doi:10.3389/fvets.2022.888207)
Supplement: Supplementary file 1 [file Data_Sheet_1.ZIP › Supplementary file/miRDeep2 file.zip!/miRDeep2 file/Know_Structure/LP42A2/oar-mir-134.pdf]

miRBase precursor : oar-mir-134
Total read count : 115
oar-miR-134-5p read count : 106
oar-miR-134-3p read count : 8
remaining reads : 1

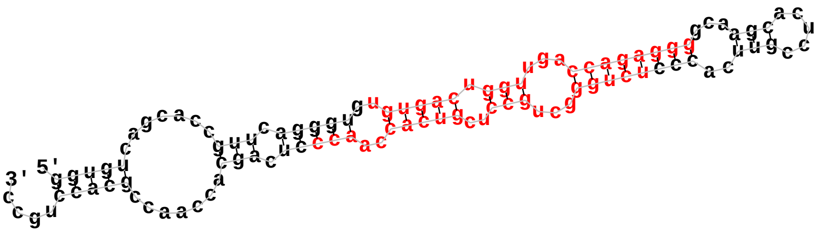

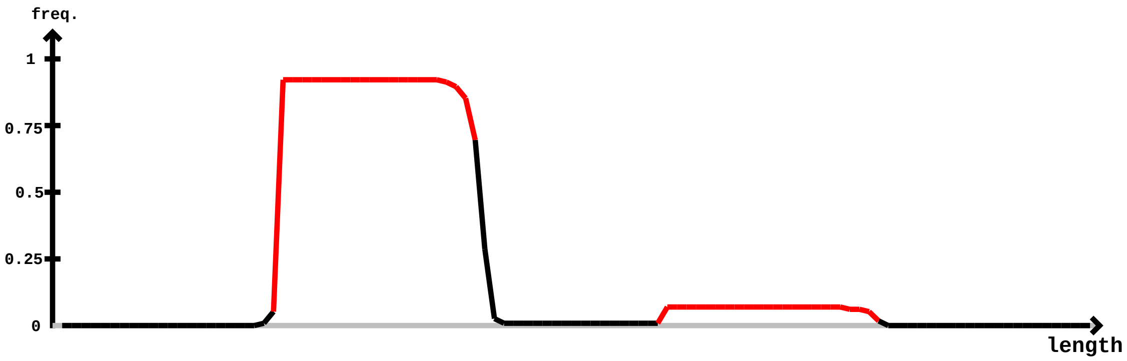


```
                                                                      oar-miR-134-3p
                         oar-miR-134-5p
5'- ggugucagcaccguucaggguguguacugguugaccagagggcaagcacuccguucacccucugggcugccucgucaccaacccucagcaccaaccgcaccugcc   -3'   exp
    (((((.......(((.(((((..(((((.(((...((((((((...(((.....)))..))))))))...)))..))))).))))).))).......)))))....   reads   mm   sample
    ....................ugugugacugguugaccagagg.................................................................   1   0   seq
    .....................gugugacugguugaccaga...................................................................   1   0   seq
    .....................gugugacugguugaccagagg.................................................................   4   0   seq
    ......................ugugacugguugaccag....................................................................   1   0   seq
    ......................ugugacugguugaccaga...................................................................   1   0   seq
    ......................ugugacugguugaccagag..................................................................   5   0   seq
    ......................ugugacugguugaccagagg.................................................................   13   0   seq
    ......................ugugacugguugaccagaggg................................................................   48   0   seq
    ......................ugugacugguugaccagagggg...............................................................   30   0   seq
    ......................ugugacugguugaccagaggggc..............................................................   2   0   seq
    ...........................................gcaagcacuccguucacc..............................................   1   0   seq
    .............................................................cucugggcugccucgucaccaa........................   1   0   seq
    ..............................................................ucugggcugccucgucacc..........................   1   0   seq
    ..............................................................ucugggcugccucgucaccaac.......................   4   0   seq
    ..............................................................ucugggcugccucgucaccaacc......................   2   0   seq
```
